# Effect of Diuretic Use on 30-Day Postdialysis Mortality in Critically Ill Patients Receiving Acute Dialysis

**DOI:** 10.1371/journal.pone.0030836

**Published:** 2012-03-14

**Authors:** Vin-Cent Wu, Chun-Fu Lai, Chih-Chung Shiao, Yu-Feng Lin, Pei-Chen Wu, Chia-Ter Chao, Fu-Chang Hu, Tao-Min Huang, Yu-Chang Yeh, I-Jung Tsai, Tze-Wah Kao, Yin-Yi Han, Wen-Chung Wu, Chun-Cheng Hou, Guang-Huar Young, Wen-Je Ko, Tun-Jun Tsai, Kwan-Dun Wu

**Affiliations:** 1 Division of Nephrology, Department of Internal Medicine, National Taiwan University Hospital, Taipei, Taiwan; 2 Division of Nephrology, Department of Internal Medicine, Saint Mary's Hospital, and Saint Mary's Medicine, Nursing and Management College, Yilan, Taiwan; 3 Department of Traumatology, National Taiwan University Hospital, Taipei, Taiwan; 4 Department of Surgery, National Taiwan University Hospital, Taipei, Taiwan; 5 International Harvard Statistical Consulting Company, Taipei, Taiwan; 6 Division of Nephrology, Department of Internal Medicine, Yun-Lin Branch, Douliou City, Yun-Lin County, Taiwan; 7 Department of Anesthesiology, National Taiwan University Hospital, Taipei, Taiwan; 8 Department of Pediatrics, National Taiwan University Hospital, Taipei, Taiwan; 9 NSARF: National Taiwan University Hospital Study Group on Acute Renal Failure, Taipei, Taiwan; 10 Section of Internal Medicine, Miao-Li Hospital, Department of Health, Miao-Li, Taiwan; 11 Department of Internal medicine , Min-Sheng Hospital, Tao-Yuan, Taiwan; National Taiwan University Hospital, Taiwan

## Abstract

**Background:**

The impact of diuretic usage and dosage on the mortality of critically ill patients with acute kidney injury is still unclear.

**Methods and Results:**

In this prospective, multicenter, observational study, 572 patients with postsurgical acute kidney injury receiving hemodialysis were recruited and followed daily. Thirty-day postdialysis mortality was analyzed using Cox's proportional hazards model with time-dependent covariates. The mean age of the 572 patients was 60.8±16.6 years. Patients with lower serum creatinine (p = 0.031) and blood lactate (p = 0.033) at ICU admission, lower predialysis urine output (p = 0.001) and PaO_2_/FiO_2_ (p = 0.039), as well as diabetes (p = 0.037) and heart failure (p = 0.049) were more likely to receive diuretics. A total of 280 (49.0%) patients died within 30 days after acute dialysis initiation. The analysis of 30-day postdialysis mortality by fitting propensity score-adjusted Cox's proportional hazards models with time-dependent covariates showed that higher 3-day accumulated diuretic doses after dialysis initiation (HR = 1.449, p = 0.021) could increase the hazard rate of death. Moreover, higher time-varying 3-day accumulative diuretic doses were associated with hypotension (p<0.001) and less intense hemodialysis (p<0.001) during the acute dialysis period.

**Background and Significance:**

Higher time-varying 3-day accumulative diuretic dose predicts mortality in postsurgical critically ill patients requiring acute dialysis. Higher diuretic doses are associated with hypotension and a lower intensity of dialysis. Caution should be employed before loop diuretics are administered to postsurgical patients during the acute dialysis period.

## Introduction

Postoperative acute kidney injury (AKI) is a serious complication resulting in prolonged hospital stays and high mortality rates [1]. AKI develops in 5% to 30% of postsurgical patients and is associated with a mortality rate of 60% to 90% [2], [3], [4]. Prerenal azotemia and ischemic acute tubular necrosis (ATN) are the predominant causes of AKI [5]. During the perioperative period, fluid balance is one of the most important issues [6]. It is reasonable to consider that diuretic use could help preserve urine output and thereby shorten the dialysis period for better fluid management. Loop diuretics may convert oliguric into nonoliguric form of AKI, allowing easier fluid and/or nutritional support for the patient. Furosemide is a loop diuretic and a vasodilator, which may decrease the metabolic work of the thick ascending limb [7]. Diuretic use in critically ill patients with acute kidney injury is reported not related to mortality [6]. High-dose furosemide (35 mg/kg/d orally) was used in patients with AKI requiring dialysis but did not have an impact on the survival and renal recovery rates [8]. It was reported diuretic might confer a survival advantage in patients on hemodialysis [9]. Loop diuretic use after dialysis in DOPPS study ranged from 9.2% in the United States to 21.3% in Europe, whereas use within 90 days of starting dialysis therapy ranged from 25.0% in the United States to 47.6% in Japan [9]. Preoperative diuretic use could be associated with postoperative AKI [10]; however, the association between patients' mortality and diuretic use in postoperative critical patients receiving acute dialysis has not been elucidated.

The concern about acute dialysis-related mortality usually focuses on the severity of the predialytic disease. However, predialysis risk factors may have different effects on short- and long-term survival, and the risk factors assessed at initial dialysis may change over time [11], [12]. Thus, in view of the complex relationship between diuretic use and mortality in critically ill patients, we used a comprehensive approach with both time-independent and time-dependent predictor variables to test the hypothesis that diuretic use during acute dialysis affects mortality in postsurgical patients.

## Results

### Patients' basic demographic characteristics

**Figure 1:**
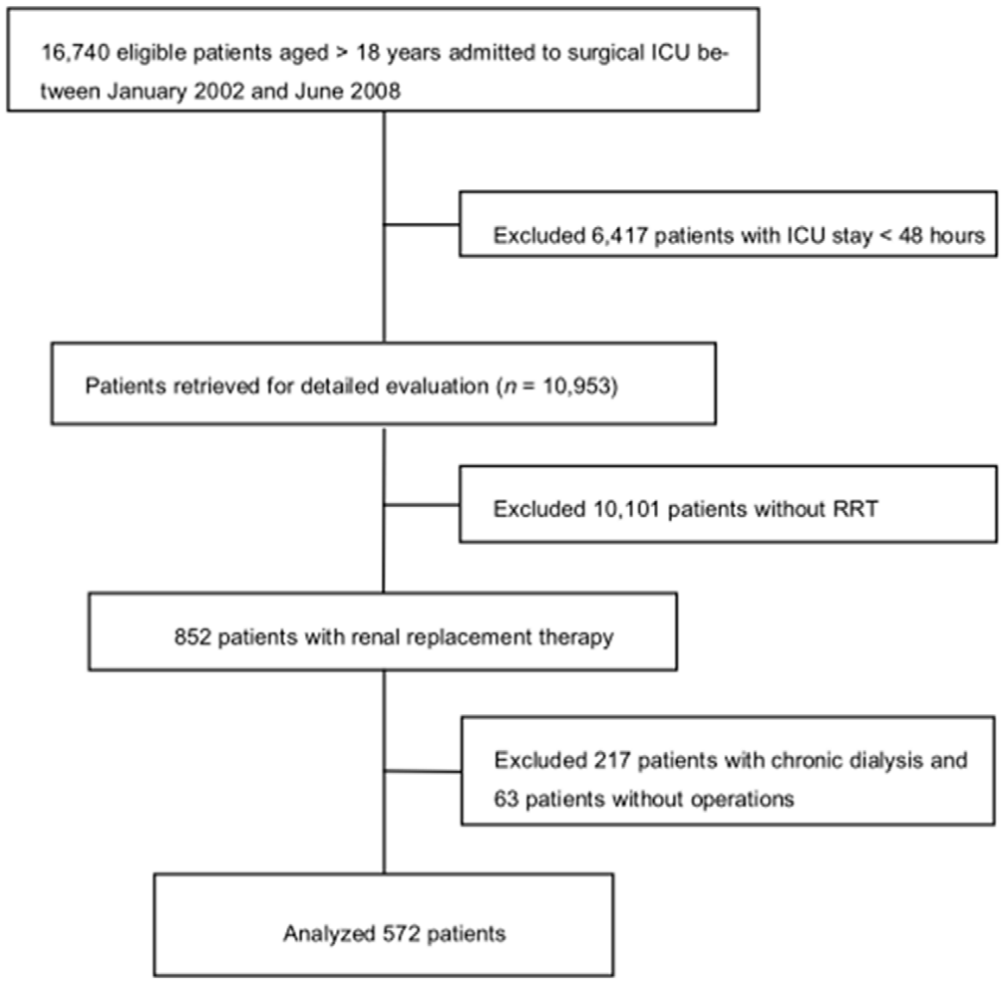
Flow diagram of the study population.

**Figure 2:**
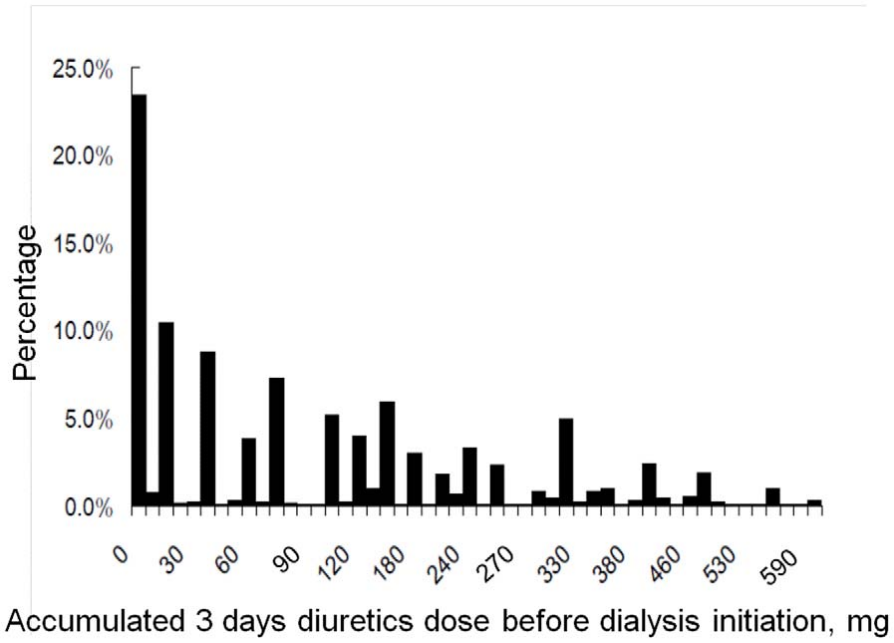
Histogram of accumulated 3-day diuretic use before dialysis initiation (equivalent of furosemide, mg).

A total of 572 critically ill patients (191 women; mean age, 60.8±16.6 years) who had received acute RRT after major surgical operations were recruited and followed daily (Figure 1). The mean APACHE II score was 11.3±6.0, and SOFA score was 8.3±3.5 at ICU admission. Among the patients, 424 (74.1%) used diuretics during the first three days of initial hemodialysis. The histogram of initially accumulated 3-day diuretic dose after RRT is shown in Figure 2. At dialysis initiation, the mean APACHE II and SOFA scores were 12.9±6.3 and 11.4±3.7, respectively. The mean time to dialysis from ICU admission was 6.6±18.0 days. Postoperatively, 283 (49.5%) patients died within 30 days after dialysis, which accounted for 77.3% of hospital mortality. After dialysis, only 193 (33.7%) patients were successfully withdrawn from acute dialysis.

### Predialysis assessments

Before dialysis, 417 (72.9%) postsurgical patients received inotropics treatment, and 94 (16.4%) developed anuria. Most patients underwent cardiovascular surgery (203, 35.5%). Although there were multiple indications for starting hemodialysis, oliguria (51.6%) and fluid overload (15.7%) were the leading causes for acute hemodialysis. Most (73.6%) of the patients had a status that was more severe than the injury criteria of the RIFLE classification upon initial dialysis.

The indications of acute hemodialysis were the same between 3-day predialysis diuretic and nondiuretic use groups. The SOFA score was higher at ICU admission in the nondiuretic than the diuretic group (*p* = 0.006). However, there were no differences in duration from hospital admission to dialysis, duration from ICU admission to dialysis, comorbidities, operation types, indication of acute dialysis or disease severity at the beginning of dialysis between the two groups (Table 1, 2). Patients with diuretic use had lower PaO_2_/FiO_2_ (*p* = 0.011) before the first hemodialysis session.

**Table 1:** Comparison of demographic of the nondiuretic and diuretic groups.

	Nondiuretic	Diuretic	*p*
	(n = 148)	(n = 424)	
Male gender	103 (69.6%)	278 (65.6%)	0.418
Age, mean (SD), years	59.8 (18.2)	61.2 (16.1)	0.386
BMI, mean (SD), Kg/M^2^	23.6 (4.1)	23.5 (3.8)	0.780
Elective operation	48 (32.4%)	170 (40.1%)	0.115
IABP	28 (18.9%)	79 (18.3%)	0.999
ECMO	40 (27.0%)	87 (20.5%)	0.108
CPR	30 (20.3%)	64 (15.1%)	0.156
**Comorbid diseases**			
Diabetes mellitus	37 (25.0%)	141 (33.3%)	0.064
Hypertension	62 (41.9%)	188 (44.3%)	0.631
CHF	5 (3.4%)	17 (4.0%)	0.999
Cirrhosis	17 (11.5%)	47 (11.1%)	0.880
CKD	87 (58.8%)	234 (55.2%)	0.501
Sepsis	35 (23.6%)	87 (20.6% )	0.485
**Systemic organ failure**			
Central nervous system	28 (18.9%)	87 (20.5%)	0.722
Respiratory	27 (18.2%)	114 (26.9%)	0.036
Cardiac	42 (28.4%)	147 (34.7%)	0.187
Liver	29 (19.6%)	61 (14.4%)	0.149
**Operation**			
Abdominal	50 (33.8%)	129 (30.5%)	0.696
Cardiovascular	76 (51.4%)	207 (48.9%)	
Chest	14 (9.5%)	54 (12.8%)	
Neurology	4 (2.7%)	22 (5.2%)	
Urology	3 (2.0%)	8 (1.9%)	
Orthopedic	1 (0.7%)	3 (0.7%)	

NOTE. Data are no. (%) of patients, unless otherwise indicated.

**Abbreviations:** BMI, body mass index; CHF, congestive heart failure; CPR, cardiopulmonary resuscitation; CKD, chronic kidney disease; ECMO, extracorporeal membrane oxygenation; IABP, intraaortic balloon pump; SD, standard deviation.

§ Data correspond to the worst value in the 24 hours preceding the time point.

**Table 2:** Comparison of predialysis clinical characteristics of the nondiuretic and diuretic groups.

	Nondiuretic	Diuretic	*p*
	(n = 148)	(n = 424)	
**Indication for acute dialysis**			
Azotemia	48 (32.4%)	117 (27.6%)	0.292
Fluid overload	23 (15.5%)	67 (15.9%)	0.999
Hyperkalemia	11 (7.4%)	32 (7.5%)	0.999
Oliguria	72 (48.6%)	223 (52.6%)	0.445
Acidosis	4 (2.7%)	17 (4.0%)	0.615
**At dialysis initiation** §			
Hospital admission to dialysis, mean (SD), days	18.1 (36.7)	17.7 (35.5)	0.913
ICU admission to dialysis, mean (SD), days	4.7 (8.3)	7.3 (20.9)	0.134
Ventilator	122 (82.4%)	361 (85.1%)	0.432
NPO	89 (60.1%)	248 (58.5%)	0.771
BUN, mean (SD), mg/dL	65.5 (36.2)	64.4 (37.8)	0.750
sCr, mean (SD), mg/dL	3.4 (2.0)	3.3 (1.8)	0.831
MAP, mean (SD), mmHg	80.1 (17.4)	81.2 (16.8)	0.498
CVP, mean (SD), cm	13.8 (6.0)	14.2 (5.6)	0.560
PaO2/FiO2, mean (SD), mmHg	320.6 (209.2)	279.6 (149.4)	0.011
Urine output, mean (SD), mL/24 hours	812.5 (840.5)	700.9 (755.1)	0.134
Anuria	29 (19.6%)	65 (15.5%)	0.250
Inotropic agents	99 (66.9%)	318 (75.2%)	0.053
Lactate, mean (SD), mmol/L	5.2 (4.5)	4.5 (2.2)	0.147
Sodium, mean (SD), mmol/L	139.8 (8.1)	139.6 (7.7)	0.721
Potassium, mean (SD), mmol/L	4.25 (0.86)	4.2 (0.8)	0.906
**Disease severity score**			
APACHE II at ICU admission	11.4 (5.9)	11.2 (6.0)	0.688
APACHE II at initial dialysis	12.9 (5.5)	12.4 (6.2)	0.380
SOFA at ICU admission	9.0 (3.7)	8.1 (3.4)	0.006
SOFA at initial dialysis	10.9 (3.9)	11.2 (3.6)	0.369
SAPS at ICU admission	111.3 (14.3)	107.9 (13.4)	0.010
SAPS at initial dialysis	116.7 (13.4)	114.4 (13.6)	0.081
RIFLE at initial dialysis:			
Risk	38 (25.7%)	113 (26.7%)	0.540
Injury	28 (18.9%)	74 (17.5%)	
Failure	82 (55.4%)	237 (55.9%)	
**Dialysis modality at initial dialysis**			
IHD	34 (23.0%)	82 (19.3%)	0.540
SLED	11 (7.4%)	27 (6.4%)	
CVVH	103 (69.6%)	315 (74.3%)	

NOTE. Data are no. (%) of patients, unless otherwise indicated.

§ Data correspond to the worst value in the 24 hours preceding the time point.

**Abbreviations:** APACHE II, acute physiology and chronic health evaluation; BUN, blood urea nitrogen; CVP, central venous pressure; CVVH, continuous venovenous hemofiltration; ICU, intensive care unit; IHD, intermittent hemodialysis; MAP, mean arterial pressure; NPO, nil per os; RIFLE, risk of renal failure, injury to kidney, failure of kidney function, loss of kidney function and end-stage renal failure; SAPS, simplified acute physiological score; SD, standard deviation; SOFA, Sequential Organ Failure Assessment; sCr, serum creatinine; SLED, sustained low-efficiency dialysis.

∥ Defined as 3-day predialysis diuretic use.

At the initiation of dialysis, the survival patients had lower disease severity scores (APACHE II, SOFA, and SAPA, all *p*<0.001), but the ratio of diuretic use was the same between two groups, (*p* = 0.775) (Table 3,4).

**Table 3:** Comparison of demographic of the postdialysis 30-day survival and mortality patients.

	Survival	Mortality	*p*
	(n = 289)	(n = 283)	
Male gender	192 (66.4%)	189 (66.8%)	0.930
Age, mean (SD), years	59.7 (17.1)	62.0 (16.1)	0.100
BMI, mean (SD), Kg/M2	23.7 (3.7)	23.4 (4.1)	0.403
Elective operation	118 (40.8%)	100 (35.3%)	0.169
CPR	39 (13.5%)	55 (19.6%)	0.056
Diuretic use	213 (74%)	205(72.4%)	0.376
**Comorbid diseases**			
Hypertension	131 (45.3%)	119 (42.0%)	0.449
DM	96 (32.9%)	82 (29.3%)	0.367
Cirrhosis	29 (10.0%)	35 (12.4%)	0.427
CKD	175 (60.6%)	146 (51.6%)	0.035
CHF	11 (3.8%)	11 (3.9%)	0.999
Sepsis	41 (14.2%)	81 (28.6)	<0.001
**Systemic organ failure**			
Central nervous system	36 (12.5%)	79 (27.9%)	<0.001
Respiratory	50 (17.3%)	91 (32.2%)	<0.001
Cardiac	82 (28.4%)	107 (37.8%)	0.021
Liver	30 (10.4%)	60 (21.2%)	0.001
**Operation**			
Abdominal	86 (29.6%)	93 (33.2%)	0.045
Cardiovascular	153 (52.6%)	130 (46.4%)	
Chest	25 (8.6%)	43 (15.4%)	
Neurology	16 (5.5%)	10 (3.6%)	
Urology	8 (2.7%)	3 (1.1%)	
Orthopedic	3 (1.0%)	1 (0.4%)	

NOTE. Data are number (%) of patients, unless otherwise indicated.

**Abbreviation:** BMI, body mass index; CPR, cardiopulmonary resuscitation; CKD, chronic kidney disease; CHF, congestive heart failure; DM, diabetics mellitus; SD, standard deviation.

**Table 4:** Comparison of predialysis characteristics of the postdialysis 30-day survival and mortality patients.

	Survival	Mortality	*p*
	(n = 289)	(n = 283)	
**Indication for acute dialysis**			
Azotemia	79 (27.3%)	86 (30.4%)	0.460
Fluid overload	31 (10.7%)	60 (21.2%)	<0.001
Hyperkalemia	21 (7.3%)	22 (7.8%)	0.875
Oliguria	143 (49.5%)	152 (53.7%)	0.317
Severe acidosis	7 (2.4%)	14 (4.9%)	0.123
**At dialysis initiation** §			
IABP	37 (12.8%)	70 (24.8%)	<0.001
ECMO	47 (16.3%)	80 (28.3%)	0.001
Diuretic use∥	216 (74.7%)	208 (73.5%)	0.775
Ventilator	230 (79.6%)	253 (89.4%)	0.001
NPO	156 (54.0%)	181 (64.0%)	0.017
Serum creatinine, mean (SD), mg/dL	3.6 (1.9)	3.1 (1.9)	0.002
Blood urea nitrogen, mean (SD), mg/dL	64.4 (33.4)	65.0 (41.1)	0.841
Potassium, mean (SD), mmol/L	4.3 (0.8)	4.2 (0.8)	0.483
MAP, mean (SD), mmHg	84.5 (17.1)	77.3 (15.9)	<0.001
APACHE II, mean (SD)	10.7 (5.3)	14.4 (6.1)	<0.001
SOFA, mean (SD)	9.9 (3.2)	12.5 (3.6)	<0.001
SAPS, mean (SD)	110.5 (11.9)	119.7 (13.4)	<0.001
PaO2/FiO2, mean (SD), mmHg	306.2 (161.6)	274.1 (172.7)	0.023
**RIFLE**			
Risk	79 (27.3%)	72 (25.4%)	0.848
Injury	52 (18.0%)	5 (17.7%)	
Failure	158 (54.7%)	161 (56.9%)	
Lactate, mean (SD), mmol/L	3.9 (4.1)	5.5 (5.1)	0.001
CVP, mean (SD), mmHg	13.6 (5.5)	14.5 (5.8)	0.053
Urine output, mean (SD), mL/24 hours	768 (792)	692 (766)	0.246
**Dialysis modality at dialysis initiation**			
IHD	78 (27.0%)	38 (13.4%)	<0.001
SLED	22 (7.6%)	16 (5.7%)	
CVVH	189 (65.4%)	229 (80.9%)	

NOTE. Data are number (%) of patients, unless otherwise indicated.

**Abbreviation:** APACHE II, acute physiology and chronic health evaluation; CVP, central venous pressure; CVVH, continuous venovenous hemofiltration; ECMO, extracorporeal membrane oxygenation; IABP, intraaortic balloon pump; MAP, mean arterail pressure; NPO, nil per os; RIFLE, risk of renal failure, injury to kidney, failure of kidney function, loss of kidney function, and end-stage renal failure (RIFLE) classification; SD, standard deviation; SOFA, Sequential Organ Failure Assessment; SAPS, simplified acute physiological score; IHD, intermittent hemodialysis; sCr, serum creatinine, SLED, sustained low-efficiency dialysis.

§ Data correspond to the worst value in the 24 hours preceding the time point.

∥ Defined as 3-day predialysis diuretic use.

### Propensity score model for diuretic use

To explore what types of patients would require diuretics upon dialysis initiation, we conducted a logistic regression analysis and established a propensity score model. Variables (Table 1,2) were included for inclusion in the new propensity score model.

Finally, based on the fitted logistic regression model (see Table 5), the propensity score for treatment with diuretic upon initializing acute dialysis was estimated (see File S1).

**Table 5:** Multiple logistic regression model for estimating the propensity score of diuretic exposure at acute dialysis initiation.

		Odds	95%		
Covariate	Estimate	ratio	confidence	limits	*p*
Diabetes (yes)	0.522	1.685	1.032	2.751	0.037
CPR (yes)	−0.522	0.593	0.335	1.051	0.074
Predialysis urine output (mL/day)	−0.001	0.999	0.999	1.000	0.001
Congestive heart failure (yes)	0.531	1.700	1.000	2.888	0.050
Creatinine at ICU admission (mg/dL)	−0.134	0.874	0.774	0.988	0.031
Lactate (mmol/L)	−0.049	0.952	0.910	0.996	0.033
Predialysis PaO_2_/FiO_2_ (mmHg)	−0.001	0.999	0.998	1.000	0.039

**Abbreviations**: CPR, cardiopulmonary resuscitation; OR, odds ratio; ICU, intensive care unit.

Adjusted for gender, age, body mass index, elective operation, cardiopulmonary resuscitation, extracorporeal membrane oxygenation, cardiopulmonary resuscitation, ventilator use, days from hospital admission to dialysis, NPO status, total parenteral nutrition, blood pressure, BUN, creatinine, lactate, urine output, body weight, anuria, inotropic equivalent, lactate, sodium, potassium, APACHE II at initializing dialysis, diabetes mellitus, hypertension, congestive heart failure, cirrhosis, chronic kidney disease, systemic organ failure (central nervous system, respiratory, cardiac, liver), operation categories (abdominal, cardiovascular, chest, neurology, urology, orthopedic), and indication for dialysis (azotemia, fluid overload, hyperkalemia, oliguria, acidosis).

### Factors associated with 30-day postdialysis mortality

**Figure 3:**
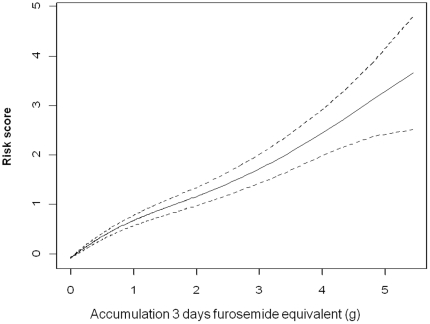
Accumulated diuretics dose predicts post dialysis mortality. Time-varying three-day accumulative diuretic dose (equivalent to furosemide dose) predicts 30 days mortality after undergoing acute dialysis. The smoothed plot of a generalized additive model included all demographic variables in the time-varying analyses, plus comorbid states (*p*<0.001).

The serial measurement of accumulated diuretics through the spectrum and duration of RRT was significant to predict 30-day postdialysis mortality (*p* = 0.021) during the dialysis period, using Cox's model adjusted with time-varying significant covariates (Table 6 and Figure 3). Regardless of diuretic use, patients who died were significantly more likely to have liver failure (*p*<0.001), and have a higher ratio of azotemia (*p* = 0.038) when dialysis began. Older age (*p*<0.001), higher APACHE II score (*p*<0.001), lower daily PaO_2_/FiO_2_ (*p*<0.001), higher daily lactate (*p*<0.001), and less daily IHD versus no dialysis (*p* = 0.030) were associated with 30-day postdialysis mortality (Table 4). The final Cox's model fit the observed data well; the adjusted generalized *R* ^2^ = 0.495.

**Table 6:** Cox's model with time-dependent covariates showing the estimated hazard ratios (HRs) for 30-day mortality in the postsurgical acute dialysis patients§.

	Parameter	Adjusted	95%	
Covariate	estimate	HR	confidence interval	*p*
**Age (years)**	0.015	1.015	1.018–1.023	<0.001
**At dialysis initiation**				
IABP	0.316	1.372	1.018–1.873	0.044
APACHE II	0.046	1.047	1.037–1.078	<0.001
**Systemic organ failure**				
Sepsis	0.432	1.541	1.183–2.015	0.001
Liver	0.699	2.011	1.476–2.763	<0.001
**Indication for acute dialysis**				
Azotemia	0.273	1.314	1.025–1.705	0.038
**Time-varying hazard (daily)**				
PaO2/FiO2 (mmHg)	−0.002	0.998	1.007–1.009	<0.001
Lactate (mmol/L)	0.171	1.186	1.144–1.236	<0.001
Time-varying three day accumulative	0.371	1.449	1.060–1.981	0.021
diuretic dose (g/3day)				
**Varying daily dialysis modality**				
Daily IHD vs. No dialysis	−0.420	0.657	0.453–0.960	0.030
Daily CVVH vs. No dialysis	0.222	1.248	0.934–1.684	0.141
Daily SLED vs. No dialysis	−0.430	0.651	0.397–1.106	0.107
Inotropic equivalents at ICU admission∥	−0.002	0.998	0.990–1.001	0.107
Inotropic equivalent at Dialysis∥	0.003	1.003	1.000–1.010	0.357
**Propensity score**				
**adjusted diuretic use**	−0.866	0.421	0.153–1.159	0.092

NOTE. Data are number (%) of patients, unless otherwise indicated.

**Abbreviations:** APACHE, acute physiology and chronic health evaluation; BUN, blood urea nitrogen; CVVH, continuous venovenous hemofiltration; IABP, intraaortic balloon pump; MBP, mean blood pressure; IHD, intermittent hemodialysis; TPN, total parenteral nutrition; SLED, sustained low-efficiency dialysis.

§ Adjusted for gender, age, body mass index, elective operation, cardiopulmonary resuscitation, extracorporeal membrane oxygenation, cardiopulmonary resuscitation, ventilator use, days from hospital admission to dialysis, NPO status, total parenteral nutrition, time-varying variables (blood pressure, BUN, creatinine, lactate, urine output, body weight, time-varying three day accumulative diuretic dose, daily dialysis modality and dialysis intensity), anuria, inotropic equivalent, lactate, sodium, potassium, APACHE II at initial dialysis, diabetes mellitus, hypertension, congestive heart failure, cirrhosis, chronic kidney disease, sepsis, shock, systemic organ failure (central nervous system, respiratory, cardiac, liver), operation categories (abdominal, cardiovascular, chest, neurology, urology, orthopedic), and indication for dialysis (azotemia, fluid overload, hyperkalemia, oliguria, acidosis).

∥ Inotropic equivalent = [(dopamine+dobutamine)+(milrinone×15)+[(epinephrine+norepinephrine+isoproterenol)×100] in mcg/kg/min [35], [40].

In the sensitivity test, a higher accumulated diuretic dose predicted mortality in the postsurgical critically ill noncardiac surgery patients requiring acute dialysis ( p<0.01).

### Diuretic dose associated with daily variables

**Figure 4:**
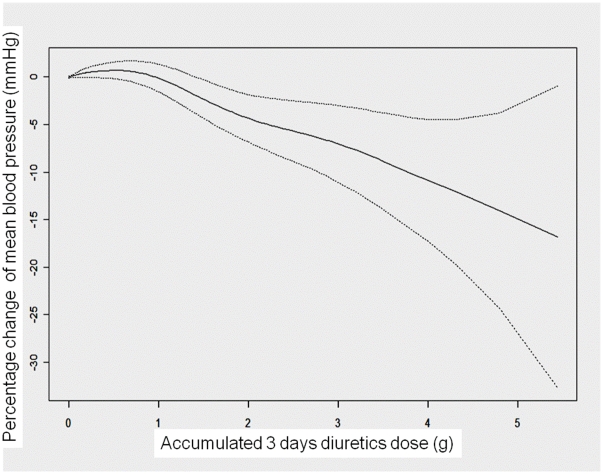
Accumulated diuretics related to blood pressure. The smoothed plot of a generalized additive model for the relationship between time-varying three-day accumulative diuretic dose and blood pressure difference in post-surgical dialysis patients with adjustments for possible linear and nonlinear effects* (*p*<0.001).

To examine the effect of diuretic use and decrease the residual confounding factors (e.g., fluid overload, oliguria, and tissue hypoxia), daily variable factors (body weight, urine output, mean blood pressure, PaO_2_/FiO_2_, lactate, BUN, and creatinine) were correlated to the time-varying three-day accumulative diuretic dose, as shown in the GEE model, and plotted by smoothed GAM plot after adjusting for all the covariates listed in Table 1. Diuretic use was significantly associated with the intensity of dialysis (*p*<0.001) (Table S1) and daily mean blood pressure (*p*<0.001) (Table S2 and Figure 4), but not with daily PaO_2_/FiO_2_ (*p* = 0.293), lactate (*p* = 0.948), BUN (*p* = 0.905), body weight (*p* = 0.449), creatinine (*p* = 0.653), and urine output (*p* = 0.075).

## Discussion

Given the high incidence of AKI in the ICU and its high morbidity, better evidence is needed to guide AKI treatment strategies. Few studies have demonstrated any material benefit of diuretic use in AKI apart from augmenting urine output, and some studies have suggested potential deleterious effects [13], [14]. Although two of the previous controlled studies found a trend toward improvement in renal function recovery rates with diuretic use [15], one cohort analysis suggested that diuretics may delay renal recovery [13]. Diuretic use has not been found to shorten the duration of AKI, reduce the need for dialysis, or improve overall outcomes [16].

Our study evaluated the relationships between diuretic use and dose with subsequent mortality after dialysis, unlike the previous report by Uchino [6], which used distinct multivariate models. The current study adds to this previous work [13] in that it identifies a group of patients, who have increased mortality and not just delayed or diminished return of renal function. Firstly, to ensure that our results were reliable, we only included postsurgical AKI patients receiving acute dialysis in surgical ICUs. In our patient cohort, all patients received dialysis; however, only 70% patients in Uchino's study received dialysis. Secondly, to show the interactions between diuretics and dialysis settings, we recorded the daily dialysis modality and settings; Uchino recorded only diuretic use at the time of acute kidney injury or dialysis initiation [6].

Furosemide is nondialysable, and retained up to 6 to 8 hours in patients with chronic renal failure; however no specific dosage adjustment of furosemide is necessary for dialysis patients [17]. It is effective for the treatment of edema associated with renal failure [18]. Therefore, diuretic use and dosage is likely to have effects on patients' outcomes. In our study, similar to a previous study on high furosemide doses for established acute renal failure [8], renal recovery rates did not differ between the diuretic and nondiuretic groups. Mehta et al. proposed that diuretic use could delay the recognition of AKI and might delay the timing of dialysis [13], which might be related to patient mortality. In our study, the duration between hospital admission to dialysis or ICU admission to dialysis did not differ among patients who were taking or not taking diuretics at initial dialysis, and no lead-time bias was found between the two groups. Our propensity score, lower predialysis PaO_2_/FiO_2_ saturation, reduced urine output, lower creatinine level, and lower lactate level at admission were predictors of diuretic use, which reflected the general features of the use of diuretics in critical care.

Using a time-dependent Cox's regression model that considers risk factors changing over time, rather than a traditional Cox's model with only fixed baseline risk factors, was an important analysis strategy, especially in critical patients with changing daily variables and inconsistent diuretic use [11]. Diuretic use when dialysis was initiated was not associated with patient mortality; however, higher varying accumulated doses during the dialysis period were associated with higher mortality. The total dose of diuretics required to achieve the treatment goal might reflect the severity of renal impairment and patients' underlying clinical status. Accumulated diuretic dose was significantly associated with hypotension and mortality in a dose dependent manner. Compared with patients who were not taking diuretics, those taking diuretics had significant hypotension. (Table S2). This study produced a novel finding that patients taking diuretics had lower blood pressure in a dose-response manner, leading to a poor prognosis throughout the duration of RRT [19]. Nevertheless, the intensive high dose diuretic therapy should be tailored on the basis of a close assessment of baseline hemodynamic data and hemodynamic response to the medications, in addition to the careful diuretic dose titration and cautious evaluation of risk/benefit ratio [20].

Moreover, patients using diuretics during dialysis periods showed borderline increasing daily urine output (p = 0.075). ICU care providers may underestimate the severity of renal injury when urine output is sustained [13]. Our study has shown that loop diuretics have negative effects on the natural history of AKI, apart from a mild increase in urine output in the postsurgical critically ill group. Fluid overload was independently associated with mortality in patients with AKI [21]. However, the daily urine output and body weight in our study were not associated with diuretic dose or mortality in our cohort of dialysis patients. Fluid overload, in terms of oliguria and increased body weight, led to more intense dialysis, according to clinical adjustments made during the dialysis period (Table S1). Large doses of furosemide activate the renin-angiotensin system and sympathetic nervous system and aggravate left ventricular pump deterioration [22]. Actually, there is a risk that a tubular or glomerular injury can be generated and that a preexisting renal dysfunction can be aggravated, especially when excessive doses of loop diuretics are being erroneously administered [20]. Hence, caution should be taken when furosemide is administered to postsurgical patients undergoing dialysis.

In our report, daily dialysis with IHD demonstrated a survival benefit as compared with no dialysis; however, daily dialysis with CVVH was marginally associated with poor prognosis (Table 6). Although the results showed that CVVH had a survival disadvantage, this could reflect residual confounding by illness severity because we used CVVH in more severely, acutely ill patients. Moreover, lower dialysis intensity was related to higher accumulated diuretic use, a common observation in clinical practice. Initial clinical trial data on the use of UF have demonstrated promising cardiac outcomes with regard to fluid removal and symptom relief, without worsening renal function [23]. The addition of a solute clearance component by dialysis may provide additional benefits for these patients with varying degrees of renal impairment [24].

Moreover, our study analysis has several notable strengths. Firstly, the relationship between lower daily blood pressure and higher diuretic dose throughout the duration of RRT was a robust finding resistant to the influence of most variables. Therefore, future randomized trial studies that stratify patients according to diuretic use should stress the relationship between blood pressure and diuretic dose. Secondly, we focused on diuretic use in heterogeneous, multicenter postsurgical patients using a comprehensive statistical approach and a large prospective database of patients with acute dialysis; therefore, our results could be extrapolated to other critical care situations because the current use of diuretics depends on clinical judgment without consensus. Thirdly, the propensity score for diuretic use and the need for dialysis were adjusted for time variable models, allowing us to incorporate changes in disease progression after dialysis for AKI and potentially providing useful prognostic information regarding important complications in intensive care units. However, patient heterogeneity could lead to confounding, and most of our patients were cardiovascular in nature. Future observational studies and clinical trials with regard to AKI should attempt to understand the long-term effects of diuretic-associated episodes.

Our study has shown that higher loop diuretic doses are associated with significant grave effects on critical patients who require acute RRT after major surgery. Higher diuretic doses were associated with hypotensive episodes after initializing dialysis, lower daily oxygen saturation and lactate level or poor kidney function parameters and was associated with higher patient mortality. Forced diuresis as an indicator of adequate renal function has less scientific rationale under such circumstances. Caution should be taken when loop diuretics is administered to postsurgical patients during acute dialysis. Future larger randomized clinical trials are required to confirm and validate the adverse effect of diuretic use in acute dialysis patients.

## Methods

### Study cohort

This study was based on a clinical cohort study of the renal failure patients in the database of the National Taiwan University Hospital Study Group for Acute Renal Failure (NSARF). The Institutional Review Board of the National Taiwan University Hospital approved the study (No. 31MD03) and waived the need of informed consent because there was neither breach of privacy nor interference with clinical decisions related to patient care.

This non-concurrent prospective cohort for quality assurance has been maintained since January 2002 in one medical center (National Taiwan University Hospital, Taipei, Taiwan) and its three branch hospitals in different cities [12], [25], [26], [27], [28]. A total of 16,740 adult patients (age>18 years) were admitted to the SICU, and 852 patients received renal replacement therapy (RRT) during their hospitalization before January 2008. Patients with chronic dialysis (n = 217) and without operations (n = 63) were excluded (Fig. 1). A total of 572 patients were included in this study. Surgical procedures were considered major if the length of the ICU stay for patients in a given diagnosis-related group exceeded two days [29].

### Clinical evaluation

Demographics, clinical characteristics at ICU admission and predialysis were assessed. Clinical evaluations included medical history, physical examination, and identification of comorbid diseases. Medical history included the presence of diabetes mellitus (DM, defined as having been treated with oral hypoglycemic agents or insulin), peripheral vascular disease (defined as having had a previous vascular procedure, a history of claudication, or the presence of femoral bruits), hypertension (defined as having taken antihypertensive drugs or having systolic and diastolic blood pressures >145/95 mmHg at the time of hospitalization), and chronic kidney disease (CKD, defined as estimated glomerular filtration rate (eGFR)<60 mL/min/1.73 m^2^ noted at ICU admission) [30]. Oliguria was defined as a urine volume<100 mL in 8 hours [31]. Sepsis was defined as the presence of both infection and systemic inflammatory response syndrome (SIRS) as in previous reports [32], [33]. A diet of 1.0–1.2 g protein/kg/day was prescribed for these patients.

Critical scoring systems, hemodynamic data, biochemistry data, and urine output were assessed after SICU admission and followed prospectively from the day that hemodialysis began. Physiological calculations were performed using the worst physiologic values assessed daily. Because of severe fluctuations in hemodynamic and biochemical data [34], we recorded patients' clinical parameters such as blood pressure, BUN, lactate, creatinine, urine output, body weight and dialysis intensity daily at 8:00 a.m.

The 30-day mortality, defined as death within 30 days after acute dialysis initiation, was the primary outcome variable. Vital signs, hemodynamic data, and laboratory data between groups were examined at the time of dialysis initiation. Anuria was defined as urine output of less than 100 mL/day. Diuretics were prescribed according to the clinical judgment in terms of patients' urine output and fluid status. Therefore the dose of diuretic was collected retrospectively. Diuretics were held if daily urine output is still less than 100cc after a single dose challenge of furosemide. The daily diuretic dose of each patient was recorded and calculated after hemodialysis was initiated. Only two types of loop diuretic, bumetanide and furosemide, were used in our unit. For the calculation, 1 mg of bumetanide was considered to be equivalent to 40 mg of furosemide [13], [26]. Because the frequency and dose of diuretic varied during dialysis, the time-varying 3-day accumulative diuretic dose was integrated to analyze the effect of diuretic use and dosage on patient mortality [11]. The time-varying 3-day accumulative diuretic dose was recorded as the total dose of diuretic received within the 3-day prior to dialysis and every 3-days after dialysis initiation.

### Renal replacement therapy (RRT)

The indication for RRT has been previously reported [25], [26], [27], namely, (1) azotemia (BUN>80 mg/dL) with uremic symptoms (165 patients); (2) fluid overload with a central venous pressure >12 mm Hg or pulmonary edema with a PaO_2_/FiO_2_<300 (90 patients); (3) hyperkalemia (serum K^+^>5.5 mmol/L) despite medical treatment (43 patients); (4) oliguria (urine amount<100 mL/8 h) with or without diuretic use (295 patients); and (5) acidosis (pH<7.2 in arterial blood gas) (21 patients).

The RRT modality was chosen according to patient hemodynamics, as previously reported [12], [25]. Continuous venovenous hemofiltration (CVVH) was used if an inotropic equivalent (IE) dose [35] of more than 15 points was required to keep systolic blood pressure (SBP) above 120 mmHg when RRT was initiated; or if SBP<120 mmHg despite inotropic agents. CVVH was performed with high-flux filters (Hemofilter, PAN-10, Asahi Kasei Medical Company, Japan) using HF 400 (Infomed, Geneva, Switzerland) and a hemofiltration flow of 35 mL/kg/hour with a blood flow of 200 mL/min. Replacement fluid was bicarbonate-buffered and was administered predilutionally at a dynamically adjusted volume to achieve the desired fluid therapy goals.

Sustained low efficiency dialysis (SLED) was operated by the same group of technicians, nephrologists, and intensive care physicians as CVVH using a previously reported standard protocol [12]. SLED was delivered for 8 hours daily, from 9:00 a.m. to 5:00 p.m., using conventional HD machines (Fresenius 4008B, Fresenius Medical Care AG, Bad Homburg, Germany) and a Fresenius F8 dialyzer (Polysulfone, Fresenius Medical Care, Taipei, Taiwan). Blood flow was 200 mL/min, and the dialysate flow was 300 mL/min. Patients were eligible for SLED if they met one of the following two criteria [12]: (1) severe fluid overload (defined as an estimated ultrafiltration >2.5 L during a single dialysis session) and a central venous pressure level >15 mmHg or pulmonary edema with PaO_2_/FiO_2_<200 mmHg despite diuretic treatment, modified from a SOFA respiratory score of 3 or 4 [36], or (2) moderately unstable hemodynamics (defined as SBP 120–140 mmHg with inotropic equivalent (IE) score 5–15 [35]). Otherwise, intermittent hemodialysis was used. Typically, most patients were treated with more than one RRT modalities during an AKI episode. Because of the use of different RRT modalities (continuous and intermittent), the dialysis intensity was defined as the number of dialysis days divided by the dialysis period and determined by consensus among the attending intensivists and nephrologists.

### Disease severity

Organ failure was classified according to the following findings [25], [26]: respiratory failure, with ventilator support; coagulopathy, platelet count ≤50×10^3^/mm^3^; central nervous system failure, Glasgow coma score ≤9; cardiac failure, signs of low cardiac output with a central venous pressure >12 mm Hg and the administration of an IE>5 points; and liver dysfunction, total bilirubin ≥2.0 mg/dL with INR>1.4. Sepsis was defined as the persistence or progression of signs and symptoms of the systemic inflammatory response syndrome with a documented or presumed persistence of infection [32].

### Statistical analysis

Statistical analyses were performed using SAS software, Version 9.1.3 (SAS Institute Inc., Cary, NC, U.S.A.) and R software, Version 2.8.1 (Free Software Foundation, Inc., Boston, MA, U.S.A.). A two-sided *p* value≤0.05 was considered statistically significant. The continuous variables were summarized as mean ± standard deviation (SD) unless otherwise indicated, whereas the categorical variables were presented as proportions. A two-sample student's *t*-test was used to test the difference in the means of continuous variables between groups, and the chi-square test or Fisher's exact test was used to analyze the associations between two categorical variables.

Moreover, because diuretic use was randomly assigned, the potential selection bias was controlled by applying propensity score analysis (PSA) [37]. To estimate each patient's propensity score for diuretic use, we fitted a separate multivariable logistic regression model with the factors predicting diuretic use [6] (further seen at File S1).

In the multivariate analysis, Cox's regression model with time-dependent covariates [11] was used to test the associations between the prognostic factors and the hazard rate of mortality within 30 days. Patients were censored at the time of withdrawal from RRT or at the end of the 30-day observation period. The baseline values for mean arterial pressure, O_2_ index, IE, lactate level, BUN, creatinine, and urine output when dialysis began and the time-varying values of their repeated measurements during the 30 days after RRT were also analyzed. Besides, the frequency and modality of RRT were also added as independent covariates to evaluate the effect of RRT intensity.

Additionally, to visualize the potential nonlinear effects of continuous covariates, such as the 3-day cumulative diuretic dose, a generalized additive models (GAM)-type approach was applied to the Cox's proportional hazards model, with the aid of the loess regression and spline smoothing techniques [38].

Finally, to examine the effect of diuretic use on various time-dependent variables, marginal linear regression models were fitted to these repeatedly measured responses using the generalized estimating equations (GEE) method [39] (further seen at File S1). The estimated propensity score of diuretic exposure and the need for dialysis was also added into the GEE marginal linear regression models as a covariate to adjust for the selection bias from diuretic use [6].

## Supporting Information

File S1 **Statistics about Propensity score, Regression model and Generalized estimating equation (GEE) model.**
(DOCX)

Table S1 **Diuretic dose and dialysis intensity.** Generalized estimating equation (GEE) model after adjusting propensity score including diuretic dose and significant time-dependent covariates was used to evaluate intensity of daily dialysis through the spectrum and duration of dialysis.
(DOC)

Table S2 **Diuretic dose and daily blood pressure.** Generalized estimating equations (GEE) model after adjusting propensity score including diuretic dose and significant time-dependent covariates was used to evaluate daily blood pressure through the spectrum and duration of dialysis.
(DOC)
