# Supplementary material for: Effect of Diuretic Use on 30-Day Postdialysis Mortality in Critically Ill Patients Receiving Acute Dialysis
Source: PLoS One. 2012 Mar 14;7(3):e30836. doi: 10.1371/journal.pone.0030836 (PMC3303770; doi:10.1371/journal.pone.0030836)
Supplement: File S1 — Statistics about Propensity score, Regression model and Generalized estimating equation (GEE) model. (DOCX) [file pone.0030836.s001.docx]

**Supplementary File**

##### *Statistical analysis*

Univariate analysis of each potential prognostic factor tended to give biased results due to the under-fitting problem, and thus multivariate analysis was conducted to identify independent prognostic factors.

##### *Propensity score model for diuretic use*

To explore what kinds of patients would receive diuretic at initializing dialysis, we conducted a regression analyses and established the propensity score model. Variables (Table 1) were included for inclusion in the new propensity score maximum model.

Finally, based on the fitted logistic regression model (see Table 3), the propensity score for diuretic use at initializing acute dialysis was estimated by the following equation:

$$\text{Estimated propensity score = }\frac{1}{1+e^{-\mathbf{A}}}$$

where **A** = Intercept + (–0.0013) × predialysis PaO_2_/ FiO_2_ (mmHg)+ (–0.134) × Creatinine (mg/dL) at ICU admission + (–0.049) × Lactate (mmol /L) at ICU admission +(–0.001) × predialysis urine output (mL/day) +(0.522) × If Diabetes +(0.531) × If congestive heart failure +(–0.522) × If preICU admission CPR.

On them, the odds ratio of lower oxygen index is 0.999 (0.998-1.000) (*p* = 0.022), lower creatinine at ICU admission is 0.704 (0.582-0.851) (*p* < 0.001), and higher creatinine at initializing dialysis is 1.276 (1.052-1.549) (*p* = 0.013).

This logistic regression model had a good calibration, estimated by the Hosmer-Lemeshow goodness-of-fit test (*p* = 0.303 > 0.05). The estimated propensity score was 0.794 ± 0.102. The propensity score itself can be interpreted as the likelihood of having diuretic based on the observed array of covariates included in the model.

***Regression model***

The goal of regression analysis was to find one or a few parsimonious regression models that fitted the observed data well for outcome prediction or effect estimation. To ensure the quality of analysis results, basic model-fitting techniques for (1) variable selection, (2) goodness-of-fit (GOF) assessment, and (3) regression diagnostics were used in our regression analyses. Specifically, the stepwise variable selection procedure (with iterations between the forward and backward steps) was applied to obtain the candidate final regression model. All the univariate significant and non-significant relevant covariates listed in Table 1 were put on the variable list to be selected and the significance levels for entry (SLE) and for stay (SLS) were set to 0.15 or larger. Then, with the aid of substantive knowledge, the best final regression model was identified manually by reducing the significance levels to 0.05 corresponding to the chosen α level. Any discrepancy between the results of univariate analysis and multivariate analysis was likely due to the confounding effects of the uncontrolled covariates in the univariate analysis. The adjusted generalized *R*^2^ and the Grønnesby-Borgan GOF test were examined to assess the GOF of the fitted Cox’s proportional hazards model. Yet, the value of the adjusted generalized *R*^2^ for Cox’s proportional hazards model is usually low. Larger *p* values of the Grønnesby-Borgan GOF test indicates better fits. Nonparametric smoothing techniques were applied to detect nonlinear effects of continuous covariates. Lastly, the statistical tools for regression diagnostics such as verification of proportional hazards assumption, residual analysis, detection of influential cases, and check for multicollinearity were used to discover model or data problems. The potential multicollinearity problem was detected by computing the variance inflating index (VIF) with the value of 10 as the threshold.

***Generalized estimating equation (GEE) model***

##### To examine the effect of diuretic use on various time-dependent variables such as blood pressure, BUN, creatinine, and dialysis frequency, the marginal linear regression models were fitted to these repeatedly measured responses using the generalized estimating equations (GEE) method.[28] The GEE method helped us obtain consistent estimates of the standard errors for the estimated regression coefficients in the marginal linear regression models so that we could make consistent inferences on the regression coefficients from the correlated data based on the correct *p* values.^[28]^ In the GEE analysis, if the first-order autocorrelation (i.e., AR (1)) structure fitted the repeated measures data well, the model-based estimates of the standard errors for the estimated regression coefficients were used; otherwise, the empirical standard error estimates were reported instead.[28] Similar to the above survival analysis, the estimated propensity score of diuretic use and requiring dialysis were also added into the GEE marginal linear regression models as a covariate to adjust for the selection bias from diuretic use.[6]
