# Supplementary material for: Effect of Diuretic Use on 30-Day Postdialysis Mortality in Critically Ill Patients Receiving Acute Dialysis
Source: PLoS One. 2012 Mar 14;7(3):e30836. doi: 10.1371/journal.pone.0030836 (PMC3303770; doi:10.1371/journal.pone.0030836)
Supplement: Table S1 — Diuretic dose and dialysis intensity. Generalized estimating equation (GEE) model after adjusting propensity score including diuretic dose and significant time-dependent covariates was used to evaluate intensity of daily dialysis through the spectrum and duration of dialysis. (DOC) [file pone.0030836.s002.doc]

**Supplementary Table 1: Generalized estimating equation (GEE) model after adjusting propensity score including diuretic dose and significant time-dependent covariates was used to evaluate intensity of daily dialysis through the spectrum and duration of dialysis.^§^**

| **Covariate** | **Estimate** | **95% Confidence Limits** | | ***P*** |
| --- | --- | --- | --- | --- |
| Propensity score adjusted diuretic use | 1.716 | 1.324 | 2.109 | <.001 |
| ECMO | 0.940 | 0.788 | 1.091 | <.001 |
| Heart failure | 0.530 | 0.419 | 0.641 | <.001 |
| Liver failure | -0.524 | -0.654 | -0.394 | <.001 |
| Potassium at dialysis initiaztion (mmol/L) | -0.251 | -0.304 | -0.197 | <.001 |
| Oliguria | 0.309 | 0.218 | 0.400 | <.001 |
| **Time-varying hazards:** |  |  |  |  |
| Daily body weight (kg) | 0.010 | 0.006 | 0.014 | <.001 |
| Daily lactate (mmol/L) | 0.341 | 0.300 | 0.383 | <.001 |
| 3 day accumulated diuretic dose (g/3 days) | -0.0001 | -0.0004 | -0.0002 | <.001 |

**Abbreviation:** ECMO = Extracorporeal membrane oxygenation.

^§^ Adjusted for Adjusted for gender age, body mass index, elective operation, cardiopulmonary resuscitation, Extracorporeal membrane oxygenation, cardiopulmonary resuscitation, Ventilator use, days from Hospital admission to dialysis, nothing Per Os, total parenteral nutrition, time varying variables (blood pressure, BUN, creatinine, lactate, urine output, body weight, varying 3 day accumulated diuretic dose, anuria, inotropic equivalent, Lactate, Sodium, Potassium, APACHE II at initializing dialysis, diabetes mellitus, hypertension, congestive heart failure, cirrhosis, chronic kidney disease, organ systemic failure (Central nervous system, Respiratory, cardiac, liver), operation categories (abdominal, cardiovascular, chest, neurology, urology, orthopedics), and indication for dialysis (azotemia, fluid overload, hyperkalemia, oliguria, acidosis).
