# Supplementary material for: Effect of Diuretic Use on 30-Day Postdialysis Mortality in Critically Ill Patients Receiving Acute Dialysis
Source: PLoS One. 2012 Mar 14;7(3):e30836. doi: 10.1371/journal.pone.0030836 (PMC3303770; doi:10.1371/journal.pone.0030836)
Supplement: Table S2 — Diuretic dose and daily blood pressure. Generalized estimating equations (GEE) model after adjusting propensity score including diuretic dose and significant time-dependent covariates was used to evaluate daily blood pressure through the spectrum and duration of dialysis. (DOC) [file pone.0030836.s003.doc]

**Supplementary Table 2: Generalized estimating equations (GEE) model after adjusting propensity score including diuretic dose and significant time-dependent covariates was used to evaluate daily blood pressure through the spectrum and duration of dialysis.**

| **Covariate** | | **Estimate** | **95% Confidence Interval** | | | ***p*** | |
| --- | --- | --- | --- | --- | --- | --- | --- |
| Propensity score adjusted diuretic use | 3.600 | | | -5.633 | 12.832 | | 0.445 |
| Age (years) | -0.128 | | | -0.200 | -0.055 | | <.001 |
| History of hypertension | 2.475 | | | 0.181 | 4.768 | | 0.035 |
| History of liver cirrhosis | -7.257 | | | -10.689 | -3.825 | | <.001 |
| History of immuno-compromised | 4.572 | | | 0.496 | 8.648 | | 0.028 |
| Operation for cardiovascular disease | -5.060 | | | -7.467 | -2.652 | | <.001 |
| Operation for urology | 5.856 | | | 1.718 | 9.994 | | 0.006 |
| Pre-dialysis inotropic equivalent dose | -0.530 | | | -0.772 | -0.289 | | <.001 |
| **Time-varying hazards:** | | | | | | | |
| Daily creatinine (mg/dL) | 0.757 | | | 0.324 | 1.190 | | <.001 |
| Daily lactate (mmol/L) | -0.283 | | | -0.537 | -0.028 | | 0.001 |
| 3 day accumulated diuretic dose (g/3 day) | -1.285 | | | -2.000 | -0.571 | | <.001 |
| Daily varying CVVH vs. No dialysis | -5.062 | | | -5.587 | -4.538 | | <.001 |

**Abbreviation:** CVVH, continuous venovenous hemofiltration.

^§^ Adjusted for gender age, body mass index, elective operation, cardiopulmonary resuscitation, Extracorporeal membrane oxygenation, cardiopulmonary resuscitation, ventilator use, days from Hospital admission to dialysis, nothing Per Os, total parenteral nutrition, time varying variables (BUN, creatinine, lactate, urine output, body weight, varying 3 day accumulated diuretic dose, daily dialysis modality and dialysis intensity), anuria, inotropic equivalent, Lactate, Sodium, Potassium, APACHE II at initializing dialysis, diabetes mellitus, hypertension, congestive heart failure, cirrhosis, chronic kidney disease, organ systemic failure (Central nervous system, Respiratory, cardiac, liver), operation categories (abdominal, cardiovascular, chest, neurology, urology, orthopedics), and indication for dialysis (azotemia, fluid overload, hyperkalemia, oliguria, acidosis).
